# Dysregulated gene expression of SUMO machinery components induces the resistance to anti-PD-1 immunotherapy in lung cancer by upregulating the death of peripheral blood lymphocytes

**DOI:** 10.3389/fimmu.2024.1424393

**Published:** 2024-08-15

**Authors:** Ying Wang, Chao Sun, Mengmeng Liu, Panyang Xu, Yanyan Li, Yongsheng Zhang, Jing Huang

**Affiliations:** ^1^ Department of Laboratory Medicine, The First Hospital of Jilin University, Changchun, China; ^2^ Cancer Center, The First Hospital of Jilin University, Changchun, China; ^3^ Prenatal Diagnosis Center, Reproductive Medicine Center, The First Hospital of Jilin University, Changchun, China

**Keywords:** PD-1, resistance, Sumoylation, lymphocyte, peripheral blood

## Abstract

**Background:**

The majority of patients with lung cancer exhibit drug resistance after anti-PD-1 immunotherapy, leading to shortened patient survival time. Previous studies have suggested an association between epigenetic abnormalities such as methylation and clinical response to anti-PD-1 immunotherapy, while the role of SUMOylation in resistance to anti-PD-1 antibody immunotherapy is still unclear.

**Methods:**

Here, the mRNA expression of 15 SUMO machinery components in PBMC from lung cancer patients receiving anti-PD-1 immunotherapy were analyzed using real-time PCR. Base on the percentage change in mRNA levels, the relationship between the expression of SUMO machinery components and outcomes of anti-PD-1 immunotherapy, and the influencing factors of SUMOylation were evaluated. PBMC was treated with different concentrations of 2-D08 (a specific inhibitor of SUMOylation) *in vitro*, and analyzed the activation and the death rates of lymphocyte subsets by flow cytometry analysis.

**Results:**

A predictive method, base on the gene expression of three SUMO machinery components (*SUMO1*, *SUMO3* and *UBE2I*), were developed to distinguish non-responders to PD-1 inhibitors. Furthermore, the number of lymphocytes in peripheral blood significantly reduced in the dysregulated SUMOylation groups (the percentage change >100 or -50 ~ -100 groups). *In vitro* studies confirmed that lightly low SUMOylation level improved the activation status of T and NK lymphocytes, but extremely low SUMOylation level lead to the increased death rates of lymphocytes.

**Conclusion:**

Our findings implied that dysregulated gene expression of SUMO machinery components could induce the resistance of anti-PD-1 immunotherapy in lung cancer by upregulating the death of peripheral blood lymphocytes. These data might provide effective circulating biomarkers for predicting the efficacy of anti-PD-1 immunotherapy, and uncovered a novel regulatory mechanism of resistance to anti-PD-1 immunotherapy.

## Introduction

Lung cancer is one of the malignant diseases with high incidence and mortality (1, 2). Since most patients with advanced lung cancer lose the opportunity for surgical resection, they often require anti-tumor immunotherapy, such as anti-programmed cell death-1 (PD-1) immunotherapy (3). However, the majority of patients remained unresponsive or worsening of disease after PD-1 blockade, which is known as the resistance to anti-PD-1 immunotherapy (4). Research indicates that resistance is a common cause of shortened survival and increased mortality in patients with anti-PD-1 immunotherapy (5). Therefore, it is important to comprehend mechanisms of the resistance to anti-PD-1 immunotherapy and identify patients who may potentially benefit from therapeutic schedule.

Numerous studies on anti-PD-1 immunotherapy have shown that epigenetic aberrations may lead to the resistance to PD-1 inhibition, thereby leading to poor prognosis (6, 7). Small ubiquitin-like modification (SUMOylation) is an epigenetic modification gaining most of the research interest recently (8). During SUMOylation, small ubiquitin-like modifiers (SUMO) are covalently attached to target proteins via an enzymatic cascade that requires the sequential action of SUMO activating enzyme, SUMO conjugating enzyme and SUMO ligases (9, 10). Similar to other epigenetic modifications, the covalent conjugation of SUMO to protein substrates is a reversible modification and cleaved by SUMO specific proteases (SENPs), i.e., deSUMOylation (11). Cumulative studies have indicated that SUMOylation regulates a number of biological processes, including carcinogenesis, cell cycle progression, apoptosis and immune responses (12). To date, five categories of SUMO machinery components had been identified, including SUMO isoforms (SUMO1~4), SUMO activating enzyme (SAE1 and UBA2), SUMO conjugating enzyme (Ubc9, encoded by *UBE2I*), SUMO ligases (PIAS1, etc) and SUMO specific proteases (SENP1, 2, 3, 5, 6, 7) (9, 10). Owing to the critical roles of SUMO machinery components in maintaining the steady-state level between SUMOylated and deSUMOylated in substrate proteins, it is conceivable that altered expression of these components can lead to various diseases, particularly cancer (13). For example, in glioblastoma (GBM), SUMO machinery components are upregulated, such as SUMO activating enzyme (SAE1), SUMO conjugating enzyme (Ubc9) and SUMO specific protease (SENP1), promoting tumor progression (14). Similarly, upregulated SUMO conjugating enzyme (Ubc9) promoted transcription factor Slug SUMOylation play a crucial role in hypoxia-induced lung cancer progression (15). Recently, epigenetic drugs have been shown to reduce the resistance of certain cancer patients to PD-1 inhibitors (16). For example, inhibitors of histone deacetylase or inhibitors of DNA Methyltransferase effectively overcome the resistance to anti-PD-1 immunotherapy in breast cancer and melanoma (17, 18). However, the role of SUMOylation in the resistance to anti-PD-1 immunotherapy is still unclear.

In anti-PD-1 immunotherapy, treatment with the PD-1 blockade interrupted the inhibitory effect mediated by PD-1/PD-L1 axis and restored activity of lymphocytes, which are the main effector cells with an anti-tumor function (19). Recent studies have found that, during anti-PD-1 immunotherapy, only a small fraction of tumor-infiltrating lymphocytes can specifically recognize and attack cancer cells to achieve therapeutic effects (20, 21). When they are exhausted, the continuous recruitment of a large number of peripheral blood lymphocytes into tumors for supplementing tumor infiltrating lymphocytes may be important for subsequent anti-PD-1 treatment responsiveness (22, 23). At present, the number of peripheral blood lymphocytes has been adopted as a clinical prediction tool for the therapeutic efficacy of anti-PD-1 immunotherapy (24, 25). Therefore, analyzing the factors that cause changes in peripheral blood lymphocyte count during anti-PD-1 immunotherapy may help to understand the mechanism of the resistance to anti-PD-1 immunotherapy and improve treatment efficacy.

In this study, we focused attention on the mRNA expression of 15 SUMO machinery components, including four SUMO genes (*SUMO1~4*), two SUMO activating enzyme genes (*SAE1* and *UBA2*), a SUMO conjugating enzyme gene (*UBE2I*), two SUMO ligase genes (*PIAS1* and *PIAS2*) and six SUMO specific protease genes (*SENP 1*,*2*,*3*,*5*,*6*,*7*), in peripheral blood mononuclear cells (PBMC). By analyzing these genes of 105 patients with lung cancer during anti-PD-1 immunotherapy, we developed a predictive method, based on the *SUMO1*, *SUMO3* and *UBE2I*, to distinguish non-responders with a progressive disease. Further analysis showed that dysregulated gene expression of SUMO machinery components was associated with decreased peripheral lymphocyte counts during the resistance process of anti-PD-1 immunotherapy in lung cancer. The *in vitro* experiments validated that dysregulated SUMOylation were one reason for inducing increased lymphocyte death. Together, these results indicated that dysregulated gene expression of SUMO machinery components may be a potential underlying cause of developing resistance to anti-PD-1 immunotherapy, and effective circulating biomarkers for predicting the efficacy of this treatment.

## Materials and methods

### Study design

Patients were enrolled from conventional treatments at the First Hospital of Jilin University, Changchun, China, from January 2023 to December 2023. For inclusion criteria: patients were diagnosed with late-stage lung cancer (stage III/IV, including local, regional and distant recurrence). The treatment regimens were determined by clinicians based on the patient’s condition. All enrolled patients received PD-1 mAb intravenously once every 3 weeks until disease progression or unacceptable toxicity. Based on Response Evaluation Criteria in Solid Tumors (RECIST) V.1.1 criteria, these patients were divided into a responder (R) group and a non-responder (NR) group. Responders were defined as a patient who achieved complete response (CR), partial response (PR) or stable disease (SD) after anti-PD-1 immunotherapy for more than 24 weeks. Non-responders were defined as a patient who had progressive disease (PD) after anti-PD-1 immunotherapy for less than 24 weeks. Patients with other malignancies or comorbidities (e.g., heart failure, severe diabetes mellitus) were excluded. The study was approved by the Human Ethics Committee of the First Hospital of Jilin University (23K155-001). All participants had signed informed consent forms before collecting samples.

### Real-time PCR

PBMC were isolated from lung cancer patients who received anti-PD-1 immunotherapy for 9 weeks by using Ficoll-Hypaque (Sigma Aldrich, USA) density gradient centrifugation. The 9th week is in line with the recommended time node for the first response assessment of tumor according to the guidelines and consensus (26–28). Total RNA was extracted from PBMC using MolPure Cell RNA Kit (YEASEN Biotech Co., Ltd, China) following the manufacturer’s instructions. cDNA synthesis was carried out by using Hifair III 1st Strand cDNA Synthesis SuperMix for qPCR (YEASEN Biotech Co., Ltd, China). The mRNA expression of 15 SUMO machinery components were analyzed by real-time PCR using the SYBR Green PCR Master Mix (YEASEN Biotech Co., Ltd, China). Considering the comparability of data, we combined the mRNA expression levels of PBMC from 10 untreated patients with lung cancer as the baseline value and defined it as 1. The relative mRNA expression levels of 15 SUMO machinery components in PBMC were calculated using the 2–ΔΔCT method. GAPDH served as an internal control. The sequences of real-time PCR primers were shown as **Supplementary Table 1**.

### Prediction model

We calculated the percentage changes in relative mRNA level of 15 SUMO machinery components in PBMC from 105 lung cancer patients as the following formula: [(express value-baseline value)/baseline value]×100%. The utilization of the percentage change formula for data normalization had been implemented in multiple studies searching for biomarkers of cancer immunotherapy efficacy (29–31). We developed a risk score base on different percentage changes in SUMO machinery components, which could be used to identify the high risk patients for developing NR. The risk score of patient was defined as 2, if the percentage change in mRNA level of SUMO machinery components in PBMC was greater than 100 times or less than -50~-100 times. The risk score of patient was defined as 1, if the percentage change in mRNA level of SUMO machinery components in PBMC was less than 100 times and greater than -50. Receiver operating characteristic (ROC) curves were used to assess diagnostic accuracy of changes in SUMO machinery components in predicting the probability of NR.

### PBMC under treatment with the SUMOylation inhibitor

To investigate the effect of SUMOylation inhibition on lymphocytes in peripheral blood, we chose 2-D08 (YEASEN Biotech Co., Ltd, China) to treat PBMC. 2-D08 is a specific inhibitor of SUMOylation that blocks the transfer of SUMO protein from the SUMO-Conjugating Enzyme (Ubc9) thioester conjugate to substrates. In the *in vitro* culture system, PBMCs isolated from 27 lung cancer patients treated with PD-1 antibody were cultured in RPMI1640 medium containing 10% fetal bovine serum (FBS; Thermo fisher scientific, USA). Along with the concentration of 2-D08 gradually increased, we observed the dose effect of SUMOylation inhibition on lymphocytes. After 24 hours of adding 2-D08, PBMCs were harvested and analyzed by flow cytometry.

### Flow cytometry analysis

Samples were tested by 10-colour/three laser flow cytometer (FACSCantoTM, BD Bioscience, USA) and analyzed by BD FACSDiva™ software (BD Bioscience, USA). The following fluorescently-conjugated antibodies were used for cell phenotypic analysis: CD45- PerCP (BD Bioscience, USA; Cat#652803), CD3-FITC (BD Bioscience, USA; Cat#349201), CD4-APC-Cy7 (BD Bioscience, USA; Cat#557871), CD8-PE-Cy7 (BD Bioscience, USA; Cat#557746), CD19-APC (BD Bioscience, USA; Cat#652804), CD56- BV510 (BD Bioscience, USA; Cat#563041), CD69-Alexa Fluor 700 (BD Bioscience, USA; Cat#560739). The death of lymphocytes was measured by staining with Annexin V-PE antibody (BD Bioscience, USA; Cat#559763). All antibodies for flow cytometry in this study were purchased from BD Biosciences. The gating strategy for cell subsets were shown in corresponding figures.

### Statistical analysis

Differences among normally distributed variables were analyzed using a Student’s t-test. For variables that were not normally distributed, a Mann-Whitney U test or Kruskal-Wallis ANOVA test was used. Statistical significance was shown as: * *P*< 0.05, ** *P*< 0.01. *** *P*< 0.001. Statistical analyses were performed using Prism 10.0 (GraphPad Prism, RRID: SCR_002798) and SPSS 27.0 statistical software package (SPSS, RRID: SCR_002865).

## Results

### The relationship between dysregulated gene expression of SUMO machinery components in PBMC and the prevalence rates of NR

**Figure 1:**
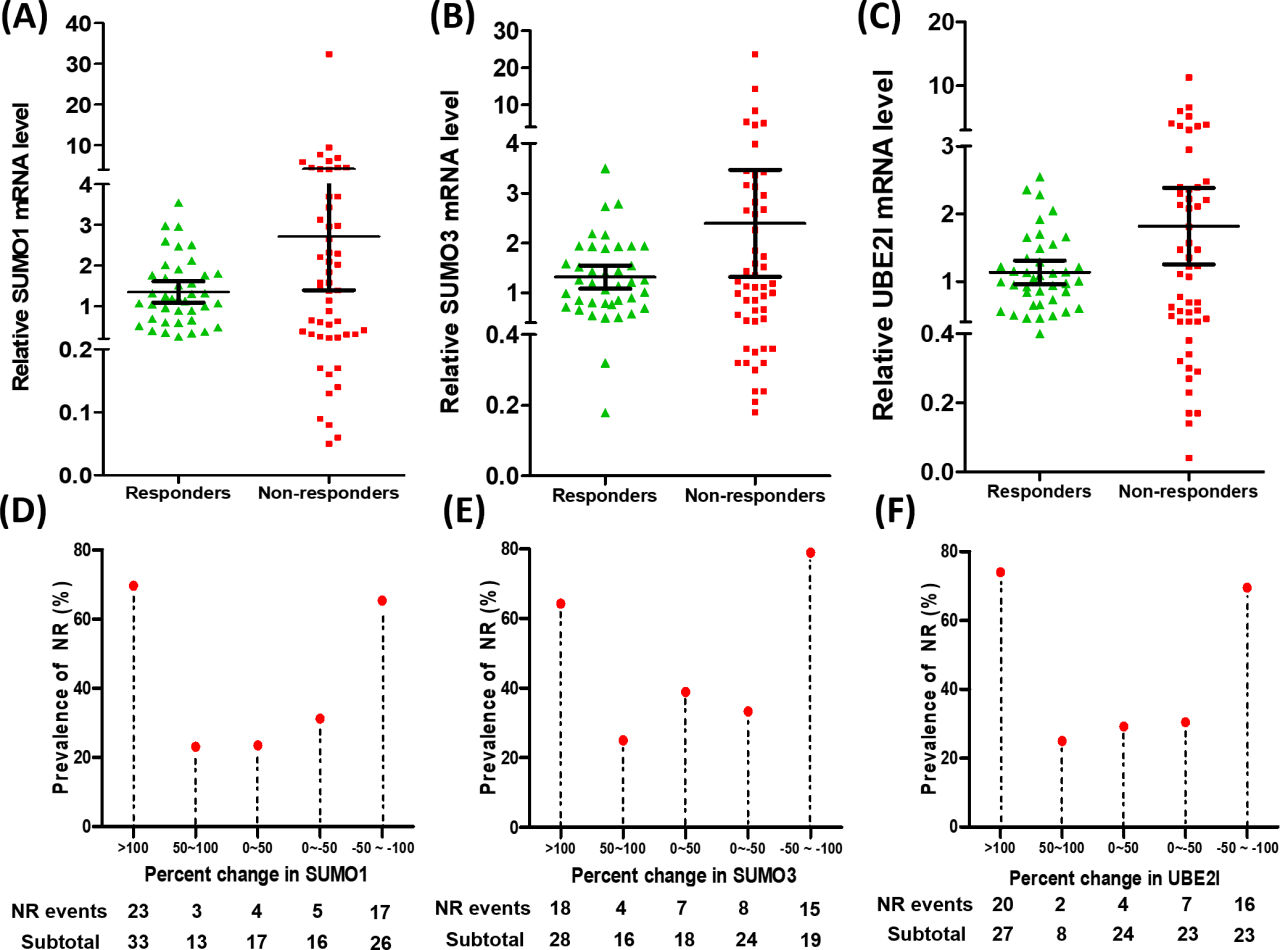
Dysregulated gene expression of SUMO machinery components in PBMC was associated with the prevalence rates of NR. **(A–C)** The relative mRNA expression of *SUMO1* **(A)**, *SUMO3* **(B)** and *UBE2I* **(C)** in PBMC from responders (n=53) and non-responders (n=52). Data were expressed as mean ± 95%CI. **(D–F)** The percentage change in the relative mRNA expression of three SUMO machinery components (*SUMO1*, *SUMO3* and *UBE2I*) was associated with the prevalence rates of NR.

To search for potential regulatory points that may influence clinical response to anti-PD-1 immunotherapy, we explored the relative mRNA expression of 15 SUMO machinery components in PBMC from 105 lung cancer patients (53 responders and 52 non-responders) for 9 weeks after anti-PD-1 immunotherapy (**Figures 1A–C**; **Supplementary Figures 1, 2**). We found that the average expression levels of each gene in PBMC was not significantly different between R group and NR group. However, compared with the R group, the dots in the scatter plot of gene expression in the NR group appeared to be more scattered. Subsequently, based on analysis of variance, the discretization of relative mRNA expression of 15 SUMO machinery components between the R and NR groups was investigated. As expected, except for *SUMO4*, *SAE1, PIAS1* and *SENP5*, the discretization of the other 11 genes in the NR group was significantly higher than that in the R group (all *p* values < 0.05).

In order to accurately evaluate the relationship between the mRNA expression of SUMO machinery components and outcomes of anti-PD-1 immunotherapy, we used the formula [(express value-baseline value)/baseline value] ×100% to calculate the relative percentage change in SUMO machinery component mRNA levels after implementation of anti PD-1 immunotherapy. The baseline value is the average mRNA expression level of corresponding SUMO machinery component in PBMCs of 10 untreated lung cancer patients. By calculating, we obtained the percentage change in relative mRNA levels and used it as an indicator of the severity of SUMOylation dysfunction (32–34). Based on the distribution of percentage change data, we roughly divided it into five groups, which are >100, 50~100, 0~50, 0~-50, -50~-100. Then the prevalence rates of NR based on specific percentage changes in relative mRNA levels were analyzed. The results showed that the prevalence rates of NR had significant differences among diverse percentage change groups of *SUMO1* (**Figure 1D**, *p*=0.001), *SUMO3* (**Figure 1E**, *p*=0.003) and *UBE2I* (**Figure 1F**, *p<*0.001). Notably, the prevalence rates of NR significantly increased in the highest percentage change group (>100) and the lowest percentage change group (-50~-100), compared with other three groups. On the contrary, the percentage change of other 12 genes had no significant correlation with the prevalence rates of NR (**Supplementary Figures 1, 2**). These results indicate that the dysregulation of *SUMO1*, *SUMO3*, and *UBE2I* expression in PBMC was positively correlated with the occurrence of NR and played an important role in the resistance process of anti PD-1 immunotherapy.

### The predictive value of the mRNA levels of *SUMO1*, *SUMO3* and *UBE2I* in PBMC for clinical response to anti-PD-1 immunotherapy

**Figure 2:**
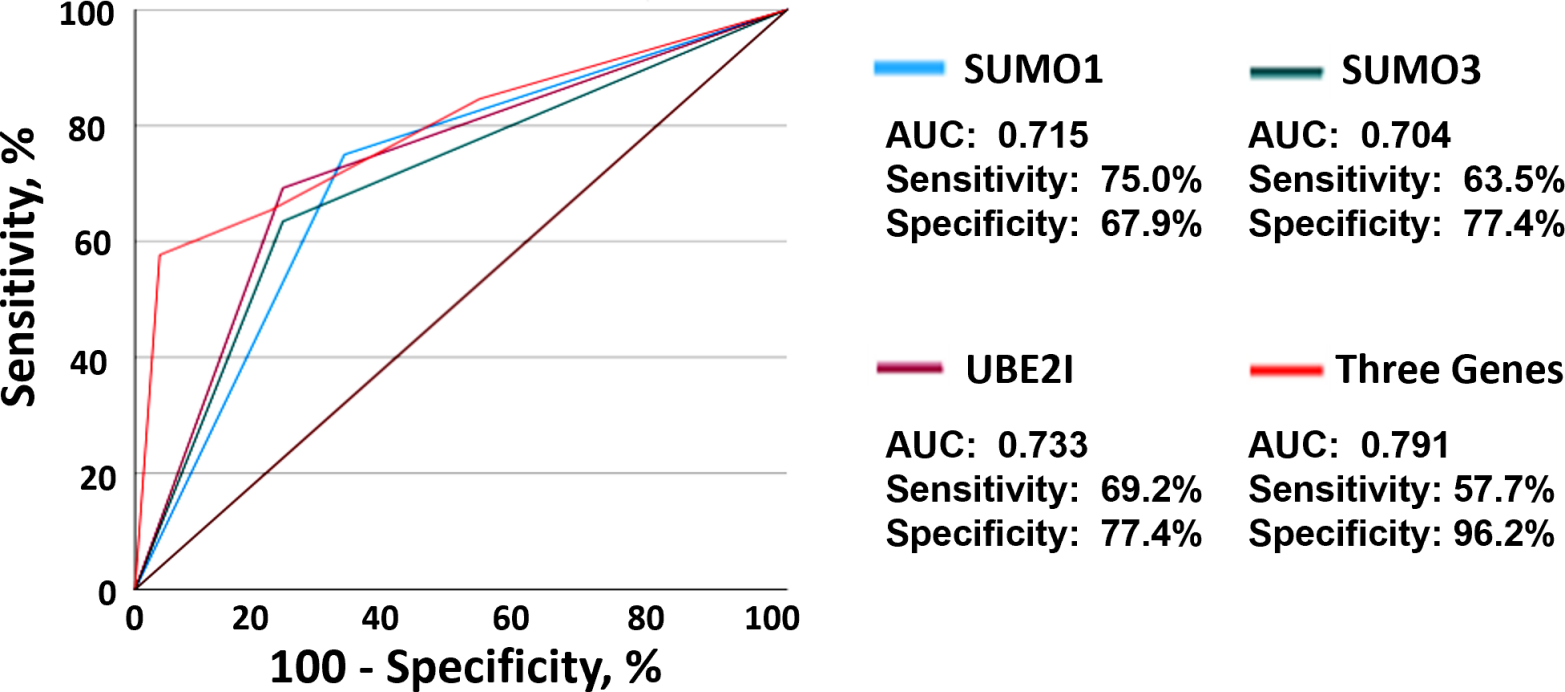
The mRNA levels of three SUMO machinery components predicted clinical response to anti-PD-1 immunotherapy. ROC analysis based on *SUMO1* (blue), *SUMO3* (green), *UBE2I* (purple) and combined three genes (red) for the diagnosis of non-responders was shown.

To evaluate the predictive ability of *SUMO1*, *SUMO3* and *UBE2I* for clinical response to anti-PD-1 therapy, we developed a risk score based on different percentage changes in mRNA level of three genes. The patients were grouped into the high-risk group and scored as 2, if the percentage change in mRNA level of three genes in PBMC was greater than 100 times or less than -50~-100 times. The patient was grouped into the low-risk group and scored as 1, if the percentage change in mRNA level of three genes in PBMC was less than 100 times and greater than -50. ROC curves was used to assess diagnostic accuracy of the percentage change in three genes to predict the probability of NR (**Figure 2**). *SUMO1* predicted the probability of NR with a sensitivity of 75.0% and specificity of 67.9% (cut-off value: 1.5; area under the curve [AUC]: 0.715; 95% confidence interval [CI]: 0.614 to 0.815; *P <*0.001). The AUC for *SUMO3* to predict NR was 0.704 (cut-off value: 1.5; 95% CI: 0.603 to 0.805; *P* < 0.001), and the sensitivity and specificity were 63.5% and 77.4%, respectively. ROC analysis revealed that *UBE2I* (cut-off value: 1.5; AUC: 0.733; 95% CI 0.635-0.831; *P* < 0.001; sensitivity: 69.2%; specificity: 77.4%) had best predictive power for the probability of NR among three genes. Moreover, the analysis of combined three genes (cut-off value, AUC, sensitivity and specificity of 5.5, 0.791, 57.7% and 96.2%, respectively) yielded a higher diagnostic accuracy than that of single gene in NR diagnosis. These results suggested that the percentage change in mRNA level of 3 SUMO machinery components (*SUMO1*, *SUMO3* and *UBE2I*), either alone or in combination, could to some extent predict clinical response to anti-PD-1 immunotherapy.

### The influencing factors of dysregulated SUMO machinery components in peripheral blood of lung cancer patients

**Figure 3:**
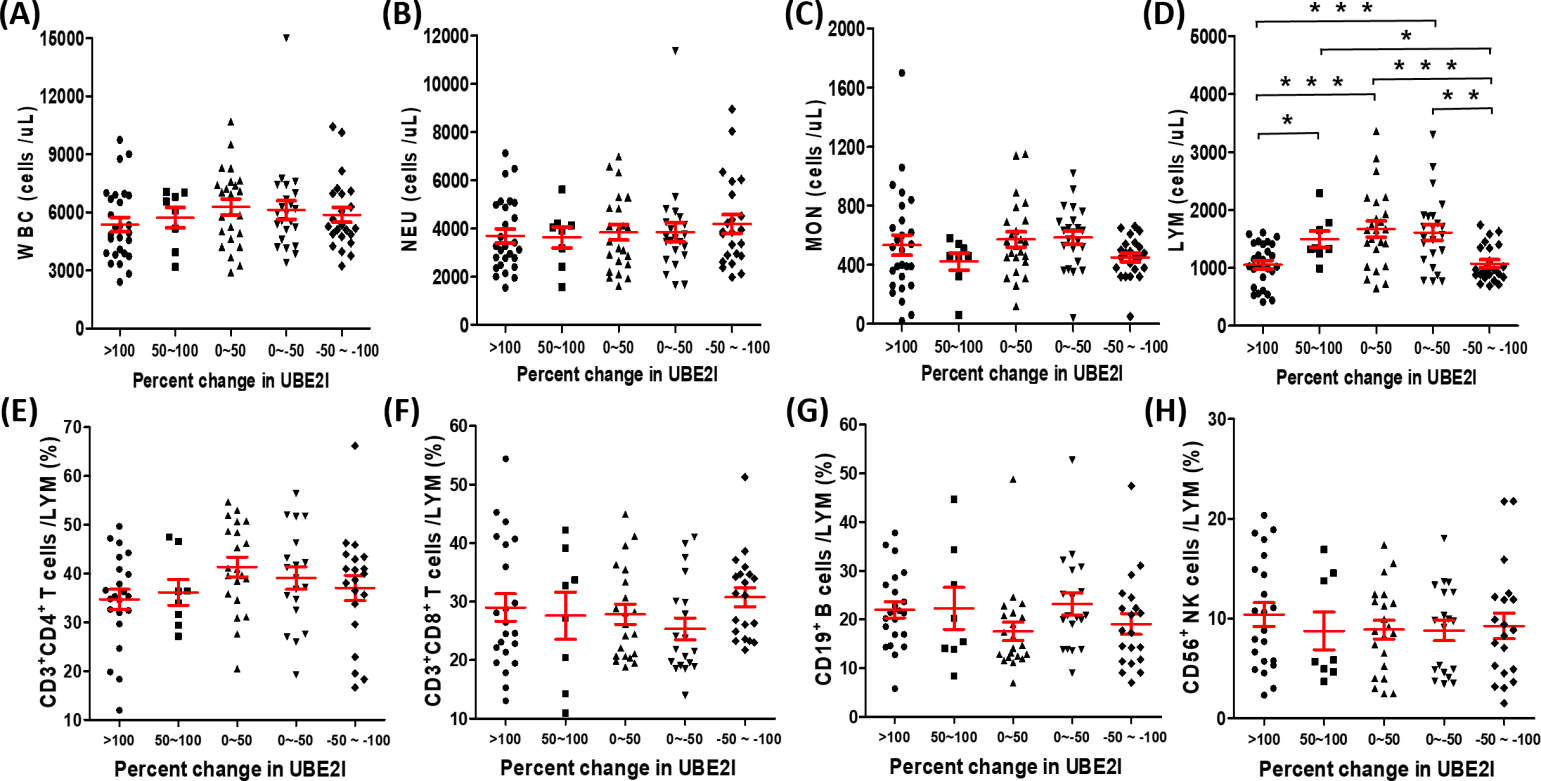
Association between the percentage change in *UBE2I* mRNA level and different white blood cell populations in peripheral blood of lung cancer patients. **(A–D)** The absolute counts of white blood cell (WBC), neutrophils (NEU), monocytes (MON) and lymphocytes (LYM) were compared among different groups base on the percentage change of *UBE2I*. **(E–H)** The percentages of lymphocyte subsets were compared among different groups base on the percentage change of *UBE2I*. Student’s paired t-test, * *P* < 0.05, ** *P* < 0.01, *** *P* < 0.001.

Due to the best predictive ability of *UBE2I* for NR among SUMO machinery components, we attempted to identify the influencing factors of SUMOylation based on the percentage change in mRNA level of *UBE2I*. The enrolled 105 patients were divided into five groups for evaluation of sociodemographic, clinical, and peripheral leukocyte characteristics. As shown in **Table 1**, the results showed that the percentage change in mRNA level of *UBE2I* was not associated with age, sex, histology, disease stage and treatment. By analyzing peripheral leukocyte characteristics, we found that the number of leukocytes (white blood cells, WBC) (**Figure 3A**), neutrophils (NEU) (**Figure 3B**) and monocytes (MON) (**Figure 3C**) had no significant differences among five groups. However, as shown in **Figure 3D**, the number of lymphocytes (LYM) were significantly reduced in the highest percentage change group (>100) and the lowest percentage change group (-50~-100). Subsequently, we analyzed the data of lymphocyte subsets from 90 patients who underwent peripheral lymphocyte subset analysis. The results showed that there was no significant differences in the proportion of various lymphocyte subsets among five groups (**Figures 3E–H**). Furthermore, based on the percentage change in mRNA level of *SUMO1* (**Supplementary Figure 3**) and *SUMO3* (**Supplementary Figure 4**), the characteristics of peripheral leukocytes were similar with *UBE2I*. Our data indicated that SUMOylation related non-response to anti-PD-1 immunotherapy might be associated with decreased peripheral lymphocyte count.

**Table 1:** Baseline characteristics and *UBE2I* expression.

Patient characteristics	The percentage change of *UBE2I*					*P Value*
	>100 n=27	50~100 n=8	0 ~ 50 n=24	0~ -50 n=23	-50 ~ -100 n=23	
**Age, years**						*0.102*
Median	60.19	66.25	62.92	62.61	64.52	
Range	51~77	48~72	44~79	50~80	43~83	
**Sex, No. (%)**						*0.552*
Male	23 (85.19)	6 (75.00)	17 (70.83)	15 (65.22)	18 (78.26)	
Female	4 (14.81)	2 (25.00)	7 (29.17)	8 (34.78)	5 (21.74)	
**Histology, No. (%)**						*0.776*
Adenocarcinoma	8 (29.63)	3 (37.50)	10 (41.67)	11 (47.82)	10 (43.48)	
Squamous cell carcinoma	13 (48.16)	3 (37.50)	11 (45.83)	6 (26.09)	10 (43.48)	
Small cell carcinoma	6 (22.21)	2 (25.00)	3 (12.50)	6 (26.09)	3 (13.04)	
**Disease stage, No. (%)**						*0.784*
c-stage III	11 (40.74)	2 (25.00)	7 (29.17)	10 (43.48)	8 (34.78)	
c-stage IV	16 (59.26)	6 (75.00)	17 (70.83)	13 (56.52)	15 (65.22)	
**Treatment, No. (%)**						*0.346*
Anti–PD-1	9 (33.33)	1 (12.50)	7 (29.17)	3 (13.04)	8 (34.78)	
Chemotherapy+ anti-PD-1	18 (66.67)	7 (87.50)	17 (70.83)	20 (86.96)	15 (65.22)	

### The regulatory effect of SUMOylation inhibition on lymphocyte activity and death

**Figure 4:**
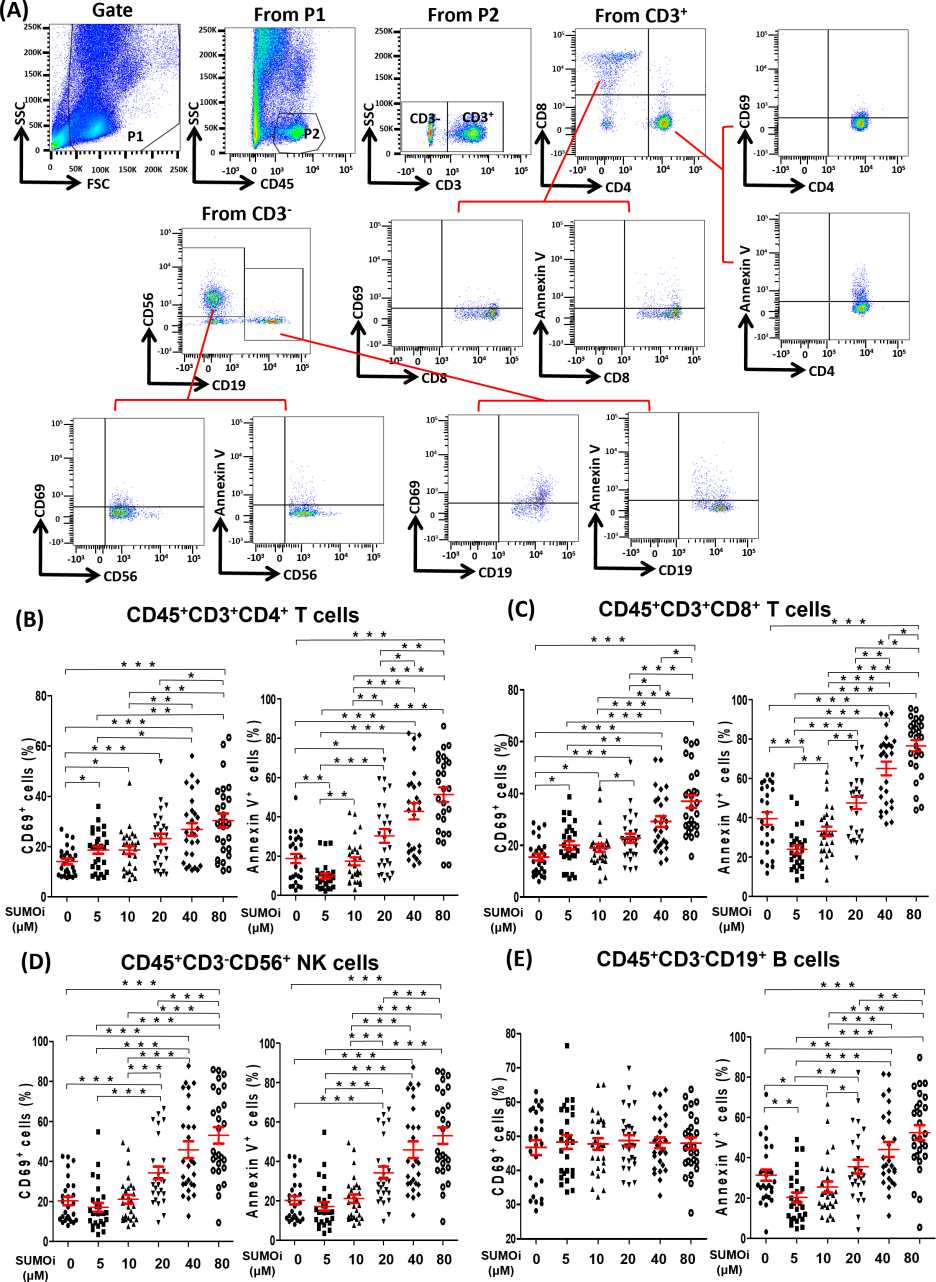
The relationship between the SUMOylation inhibition and the phenotype of lymphocyte. **(A)** Flow-cytometry dot plots showed the strategy for gating peripheral blood lymphocyte cells with CD69^+^ and Annexin V^+^ phenotype. **(B-E)** The expression of CD69 and Annexin V were analyzed on CD45^+^CD3^+^CD4^+^ T cells **(B)**, CD45^+^CD3^+^CD8^+^ T cells **(C)**, CD45^+^CD3^-^CD56^+^ NK cells **(D)** and CD45^+^CD3^-^CD19^+^ B cells **(E)**. Student’s paired t-test, * *P* < 0.05, ** *P* < 0.01, *** *P* < 0.001.

To investigate the effect of SUMOylation on lymphocytes in peripheral blood, we treated PBMC of 27 lung cancer patients receiving PD-1 treatment with different concentrations of 2-D08 (a specific inhibitor of SUMOylation) *in vitro*, and analyzed the activation status (CD69^+^) and the death rates (Annexin V^+^) of different lymphocyte subsets by flow cytometry analysis. The gating strategy was presented in **Figure 4A**. We found that the percentages of CD45^+^CD3^+^CD4^+^CD69^+^T cells (**Figure 4B**), CD45^+^CD3^+^CD8^+^CD69^+^T cells (**Figure 4C**) and CD45^+^CD3^-^CD56^+^CD69^+^NK cells (**Figure 4D**) were significantly up-regulated in a 2-D08 dose-dependent manner, but the gradually increasing concentrations of 2-D08 had no effect on the percentages of CD45^+^CD3^-^CD19^+^CD69^+^B cells (**Figure 4E**). Similarly, 2-D08 increased the percentage of CD45^+^CD3^+^CD4^+^Annexin V^+^T cells (**Figure 4B**), CD45^+^CD3^+^CD8^+^Annexin V^+^T cells (**Figure 4C**), and CD45^+^CD3^-^CD19^+^Annexin V^+^B cells (**Figure 4E**) in a dose-dependent manner at concentrations greater than 10μM. However, it was worth noting that 2-D08 reduced the percentage of these cells at 5μM concentrations (**Figures 4B, C, E**). Besides, CD45^+^CD3^-^CD56^+^NK cells also displayed an increased percentage of Annexin V^+^ cells at higher concentrations of 2-D08 (≥20μM) (**Figure 4D**). These results suggested that although inhibition of SUMOylation levels could improve the activation status of T and NK lymphocytes, extreme decline in SUMOylation levels would lead to the increased death rates of lymphocytes.

## Discussion

In this study, we found that the dysregulated mRNA expression (extremely high or low level) of three SUMO machinery components (*SUMO1*, *SUMO3* and *UBE2I*) in PBMC was related to the resistance to anti-PD-1 immunotherapy. The mRNA expression of SUMO genes (either *SUMO1* or *SUMO3*) plus the only SUMO E2 conjugating enzyme gene (*UBE2I*) was necessary for SUMOylation, and the abnormal change of their expression had a significant impact on SUMOylation level in PBMC (35). Therefore, we speculated that dysregulated SUMOylation (extremely high or low level) in PBMC may promote the occurrence of resistance to anti-PD-1 immunotherapy in lung cancer. It had been shown that up-regulation of certain SUMO machinery components correlated with the resistance in lung cancer (36). For example, *UBA2* was highly expressed in lung cancer and it knockdown increased the sensitivity of cancer cell to etoposide and cisplatin (37). *SENP1* had been reported to overexpressed in patients with lung cancer, and had a negative correlation with treatment response and could potentially predict chemosensitivity (38, 39). In spite of this, up-regulation of certain SUMO machinery components associated with favorable treatment response in some cases such as *UBE2I* 10920CG genotype enhances sensitivity to irinotecan chemotherapy in lung cancer through upregulation of *SUMO1* in tumor cells (40). These findings suggested that SUMOylation might play a complex and crucial role in the resistance of lung cancer. However, previous researchers mainly focused on the SUMOylation level during the development of resistance in tumor cells, rather than lymphocytes, which is the main effector cell in anti-PD-1 immunotherapy (41, 42). The main subpopulation of PBMC analyzed in this study is lymphocyte (43, 44). It was reported that the homeostasis and function of lymphocytes could be used to predict the response to anti-PD-1 immunotherapy in lung cancer (25). Although the steady-state level of SUMOylation played an essential role in the homeostasis and function of lymphocytes, the relationship between dysregulated SUMOylation in lymphocytes and the resistance to anti-PD-1 immunotherapy in lung cancer was still unclear (45–48).

By analyzing the immunotherapy cohort of lung cancer, we found that dysregulated gene expression of SUMO machinery components were associated with decreased peripheral blood lymphocytes counts. It was reported that the dynamically monitoring with peripheral blood lymphocytes count had great value in assessing treatment efficacy and predicting prognosis of anti-PD-1 immunotherapy (25, 49, 50). Interestingly, some researchers had found that decreased peripheral lymphocyte counts before treatment was not associated with poorer survival in patients, but persistence of decreased peripheral lymphocyte counts after 12 weeks of immunotherapy might be a poor prognostic marker of patient survival (51). However, the reason for a decreased of peripheral blood lymphocytes during therapy is unclear. Our findings evidenced that dysregulated SUMOylation is an inducing factor of decreased peripheral blood lymphocytes during anti-PD-1 immunotherapy. In *in vitro* experiments, we found that lightly low SUMOylation level (PBMC was treated with less than 5 μM 2-D08) was associated with the decreased death rates of lymphocyte, but extremely low SUMOylation level (PBMC was treated with greater than 10μM 2-D08) was associated with the increased death rates of lymphocyte. The results indicated that the steady-state level of SUMOylation might be promoting effective therapeutic response of anti-PD-1 immunotherapy by maintaining peripheral blood lymphocyte number, but dysregulated SUMOylation might be detrimental to treatment by promoting peripheral blood lymphocyte death. According to the reports, accumulation of SUMOylated STAT5 and SUMOylated Daxx resulted in growth suppression of lymphocyte (52, 53). Furthermore, in a mouse model of pancreatic ductal adenocarcinoma (PDAC), the SUMOylation inhibitor treated mice demonstrated a decrease in absolute numbers of peripheral lymphocytes. Although the SUMOylation inhibitor efficiently limited tumor growth by inhibiting cancer cell cycle progression and activating interferon signaling in lymphocytes, mouse were only well tolerated during short term treatment (54). We speculate that a strong decrease of peripheral blood lymphocyte induced by the SUMOylation inhibitor may be the main reason for the interruption of later experiments. Remarkably, the number of total lymphocyte was significantly reduced in the highest/lowest percentage change group, but there was no significant difference in the proportion of various lymphocyte subsets among five percentage change groups. This seemed to be related to the similar decreased degree of different lymphocyte subsets induced by dysregulated SUMOylation. In *in vitro* experiments, we found that SUMOylation inhibitors induced death of different lymphocyte subsets in a dose-dependent manner. Similarly, researchers found that both T and B cell development exhibited severe defects in SUMO specific protease 1 gene knockout mice (52). The above evidence suggested that dysregulated SUMOylation might lead to decrease the number of various lymphocyte subsets in a similar degree. This will result in a decrease in the number of total lymphocytes, but the proportions of various lymphocyte subsets remain unchanged.

Base on the mRNA expression of three SUMO machinery components (*SUMO1*, *SUMO3* and *UBE2I*), we displayed a predictive method to distinguish the resistance to anti-PD-1 immunotherapy. Among these three genes, *UBE2I* had the best predictive ability (AUC = 0.733) for the resistance to anti-PD-1 immunotherapy. It may be that *UBE2I*, the only SUMO conjugating enzyme gene found so far, plays a crucial role in the rate of the SUMOylation cycle (55, 56). ROC curve analysis revealed that the combined expression of *SUMO1*/*SUMO3*/*UBE2I* showed moderate prediction performance for therapeutic responses (AUC = 0.791). Recently, many biomarkers, such as single-cell RNA sequencing or non-targeted metabolomics from tumor samples, and immune infiltration in the tumor microenvironment, had been shown to be potentially useful to predict therapeutic response in lung cancer patients treated with anti-PD-1 immunotherapy (57, 58). However, local analysis of tumor tissue might be severely limited by the amount of available samples, especially since tissue would be consumed by routine molecular analyses (59). Our predictive model, either a single gene or a combination of three genes, can serve as an easily accessible circulating biomarker to evaluate anti-PD-1 treatment response, which could help clinicians to intuitively analyze initial treatment expectation.

In conclusion, our research developed a predictive method, based on the gene expression of three SUMO machinery components (*SUMO1*, *SUMO3* and *UBE2I*), to distinguish the resistance to anti-PD-1 immunotherapy. Furthermore, our findings implied that the decreased peripheral blood lymphocytes induced by dysregulated SUMOylation might be an inducing factor for the resistance to anti-PD-1 immunotherapy in lung cancer. These data might provide effective circulating biomarkers for predicting the efficacy of anti-PD-1 immunotherapy, and uncovered a novel regulatory mechanism of resistance to anti-PD-1 immunotherapy.

## Data Availability

The raw data supporting the conclusions of this article will be made available by the authors, without undue reservation.
