# Supplementary material for: Dysregulated gene expression of SUMO machinery components induces the resistance to anti-PD-1 immunotherapy in lung cancer by upregulating the death of peripheral blood lymphocytes
Source: Front Immunol. 2024 Aug 15;15:1424393. doi: 10.3389/fimmu.2024.1424393 (PMC11357960; doi:10.3389/fimmu.2024.1424393)
Supplement: Supplementary file 1 [file Image1.pdf]

Supplementary Figure 1

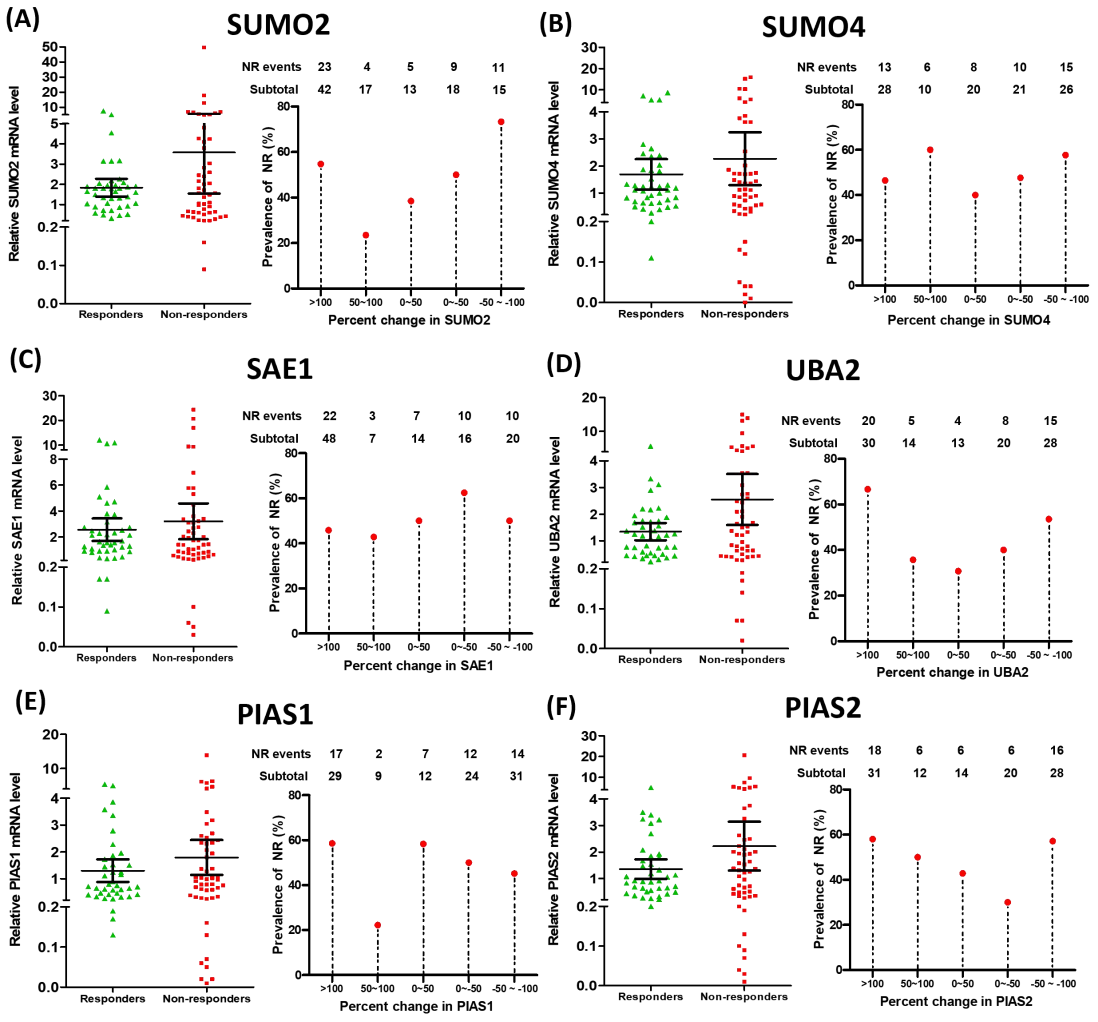

Supplementary figure 1. Association between the mRNA levels of SUMO genes, SUMO-activating enzyme genes and SUMO E3 ligase genes in PBMC and the prevalence rates of NR. (A-F) The relative mRNA expression of *SUMO2* (A), *SUMO4* (B), *SAE1* (C), *UBA2* (D), *PIAS1* (E) and *PIAS2* (F) in PBMC from responders (n=53) and non-responders (n=52) were compared. Data were expressed as mean ± 95%CI. Meanwhile, the association between the percentage changes in the relative mRNA expression of these genes and the prevalence rates of NR were analyzed.
