# Supplementary material for: Dysregulated gene expression of SUMO machinery components induces the resistance to anti-PD-1 immunotherapy in lung cancer by upregulating the death of peripheral blood lymphocytes
Source: Front Immunol. 2024 Aug 15;15:1424393. doi: 10.3389/fimmu.2024.1424393 (PMC11357960; doi:10.3389/fimmu.2024.1424393)
Supplement: Supplementary file 2 [file Image2.pdf]

Supplementary Figure 2

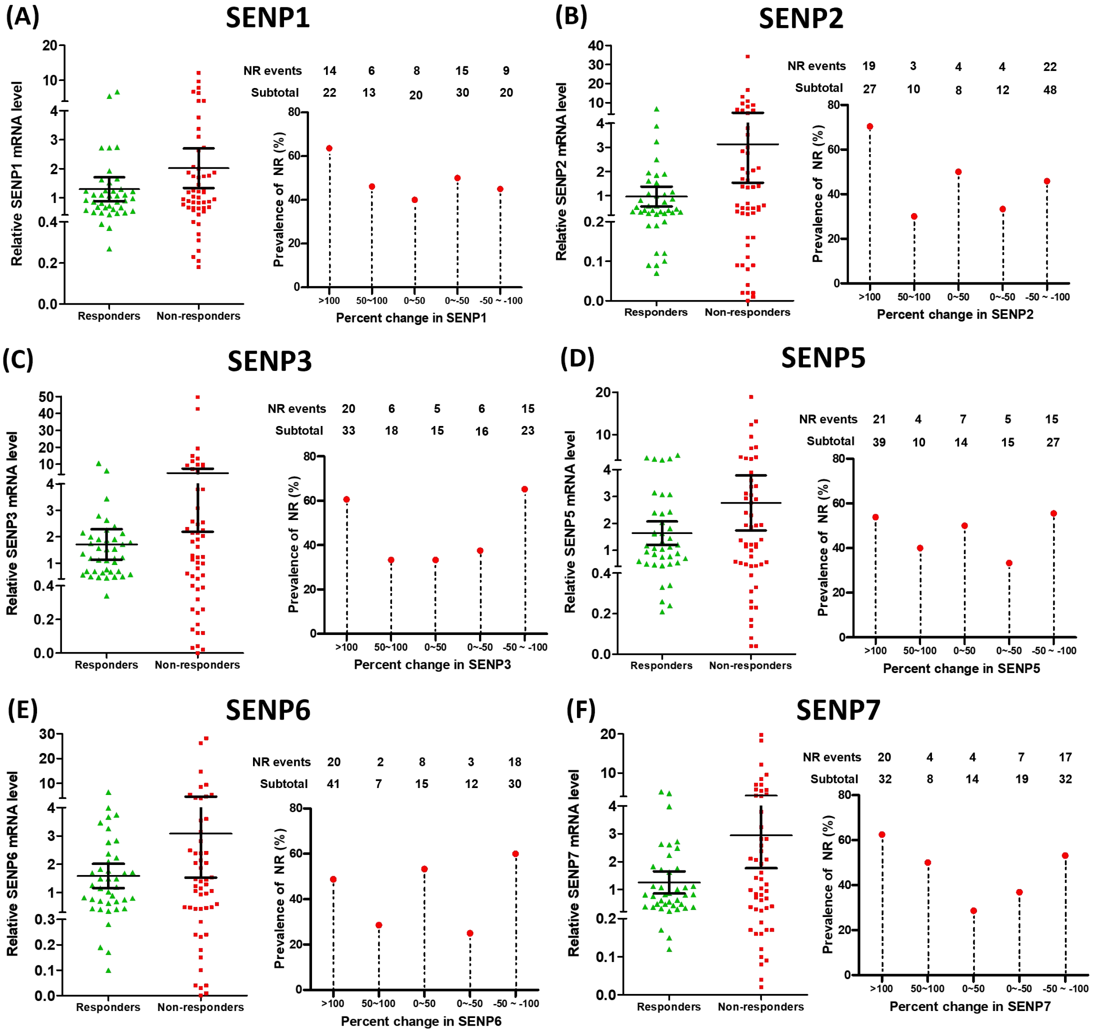

Supplementary figure 2. Association between the mRNA levels of six SUMO protease genes in PBMC and the prevalence rates of NR. (A-F) The relative mRNA expression of *SENP1* (A), *SENP2* (B), *SENP3* (C), *SENP5* (D), *SENP6* (E) and *SENP7* (F) in PBMC from responders (n=53) and non-responders (n=52) were compared. Data were expressed as mean ± 95%CI. Meanwhile, the association between the percentage changes in the relative mRNA expression of these genes and the prevalence rates of NR were analyzed.
