# Supplementary material for: Dysregulated gene expression of SUMO machinery components induces the resistance to anti-PD-1 immunotherapy in lung cancer by upregulating the death of peripheral blood lymphocytes
Source: Front Immunol. 2024 Aug 15;15:1424393. doi: 10.3389/fimmu.2024.1424393 (PMC11357960; doi:10.3389/fimmu.2024.1424393)
Supplement: Supplementary file 3 [file Image3.pdf]

Supplementary Figure 3

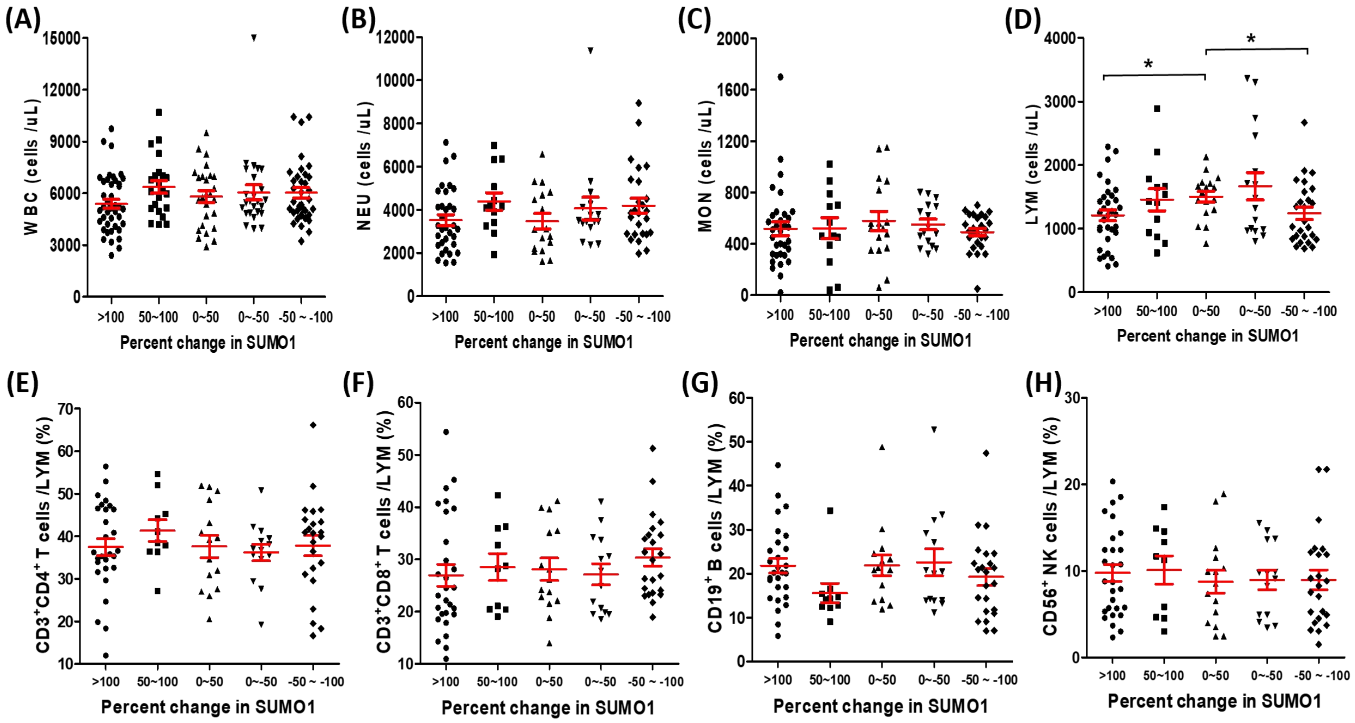

Supplementary figure 3. Association between the percentage change in *SUMO1* mRNA level and different white blood cell populations in peripheral blood of lung cancer patients. (A-D) The absolute counts of white blood cell (WBC) , neutrophils (NEU), monocytes (MON) and lymphocytes (LYM) were compared among different groups base on the percentage change of *SUMO1*. (E-H) The percentages of lymphocyte subsets were compared among different groups base on the percentage change of *SUMO1*. Student's paired t-test, * $P < 0.05$.
