# Supplementary material for: Dysregulated gene expression of SUMO machinery components induces the resistance to anti-PD-1 immunotherapy in lung cancer by upregulating the death of peripheral blood lymphocytes
Source: Front Immunol. 2024 Aug 15;15:1424393. doi: 10.3389/fimmu.2024.1424393 (PMC11357960; doi:10.3389/fimmu.2024.1424393)
Supplement: Supplementary file 4 [file Image4.pdf]

Supplementary Figure 4

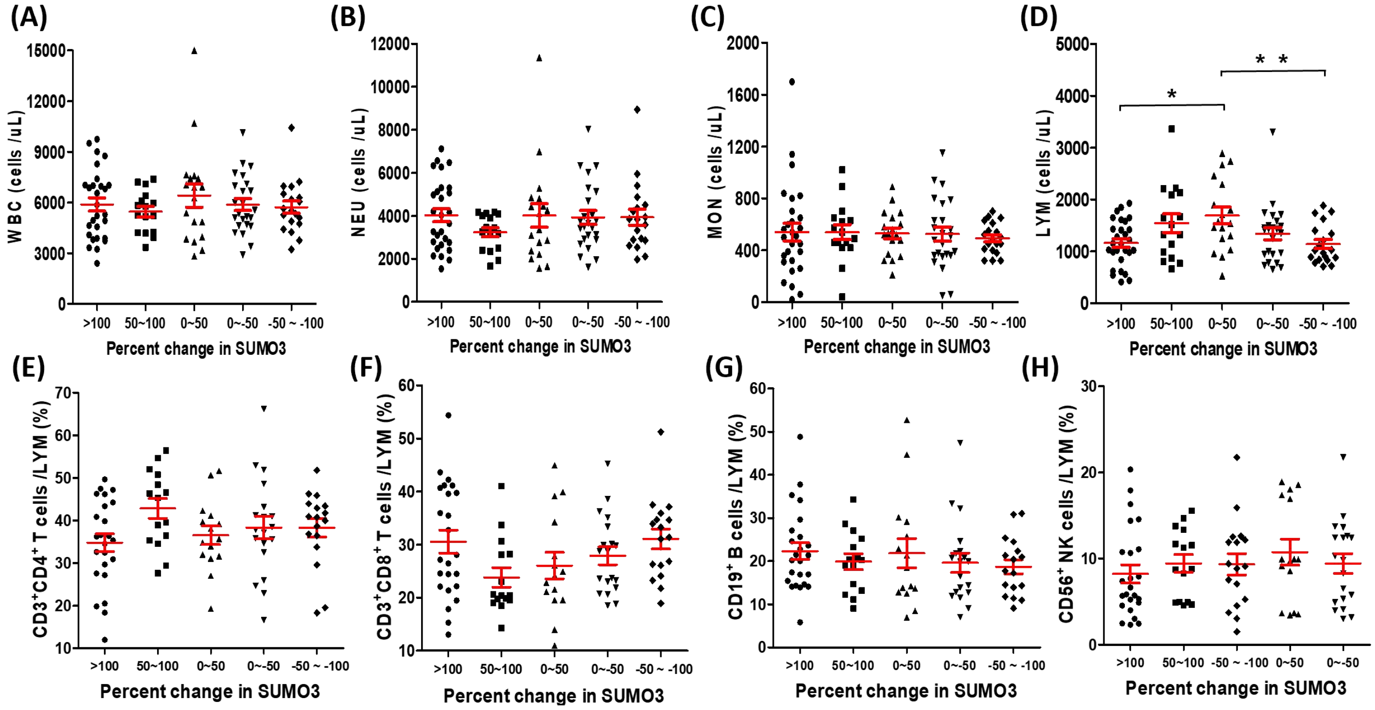

Supplementary figure 4. Association between the percentage change in *SUMO3* mRNA level and different white blood cell populations in peripheral blood of lung cancer patients. (A-D) The absolute counts of WBC, NEU, MON and LYM were compared among different groups base on the percentage change of *SUMO3*. (E-H) The percentages of lymphocyte subsets were compared among different groups base on the percentage change of *SUMO3*. Student's paired t-test, * $P < 0.05$, ** $P < 0.01$.
