# Supplementary material for: Dysregulated gene expression of SUMO machinery components induces the resistance to anti-PD-1 immunotherapy in lung cancer by upregulating the death of peripheral blood lymphocytes
Source: Front Immunol. 2024 Aug 15;15:1424393. doi: 10.3389/fimmu.2024.1424393 (PMC11357960; doi:10.3389/fimmu.2024.1424393)
Supplement: Supplementary file 5 [file Table1.pdf]

Supplementary table 1. Primer sequences for Real-time PCR.

Serial number	Gene	Forward	Reverse
**1**	GAPDH	5'- AGAAGGCTGGGGCTCATTTG -3'	5'- GTGGTCATGAGTCCTTCCAC -3'
**2**	SUMO1	5'- CGAGGTTCTGCTTACCCGAG -3'	5'- CCCCCAAGTCCTCAGTTGAA -3'
**3**	SUMO2	5'- TTGACGGGCAACCAATCAATG -3'	5'- TCAGTAGACACCTCCCGTCT -3'
**4**	SUMO3	5'- GAGAGGCAGGGCTTGTCAAT -3'	5'- CTAGAAACTGTGCCCTGCCA -3'
**5**	SUMO4	5'- CTGCTCGTGTACTCGTTAGGT -3'	5'- GACAATCCCCGTGGTTCACA -3'
**6**	SAE1	5'- GCACGACCTCCGACTACTTT -3'	5'- ACCCAGTGAGTCAAGCACATC -3'
**7**	UBA2	5'- ATCAAAGGCACAGGTTGCCA -3'	5'- TCTGGGTCGGCTTAGGATGA -3'
**8**	UBE2I	5'- GAAGTCCCGAGACAAAGGGA -3'	5'- CAGCCACGAAACCAAATGGG -3'
**9**	PIAS1	5'- AGGTAGTGAGCGGGACGTTA -3'	5'- TTTGGTTGTTCTCCATCAGCTACA -3'
**10**	PIAS2	5'- TGGCGGATTTCGAAGAGTTGA -3'	5'- GTTCGTGGATATCGGCGTCT -3'
**11**	SENP1	5'- CGGTTCCGGTTCGGACTTT -3'	5'- TCGCCTGAGCCAAGAAAACT -3'
**12**	SENP2	5'- GTGGTGGTTAAGACGGCGAA -3'	5'- TGCCGAGAATCCTAACCAGC -3'
**13**	SENP3	5'- CCGACCCTCTTTTGATGCCT -3'	5'- AGTGGAATGTCAGCGAGGTG- 3'
**14**	SENP5	5'- TTCTGTTGATTCCTATTCACCTGG -3'	5'- CAGTCTGCCAACCCTGAAGA -3'
**15**	SENP6	5'- GCCCAAAGCAGTCTGGACC -3'	5'- GTTCAACACCTTGGAGCCGA -3'
**16**	SENP7	5'- TTCAGCAGCGGGAGAAGATG -3'	5'- CAAAGGGAGAGTCCAGCGTT -3'
